# Cysticercosis and taeniasis cases diagnosed at two referral medical institutions, Belgium, 1990 to 2015

**DOI:** 10.2807/1560-7917.ES.2019.24.35.1800589

**Published:** 2019-08-29

**Authors:** Veronique Dermauw, Steven Van Den Broucke, Lieselotte Van Bockstal, Leon Luyten, Kim Luyckx, Emmanuel Bottieau, Pierre Dorny

**Affiliations:** 1 Unit of Veterinary Helminthology, Institute of Tropical Medicine, Antwerp, Belgium; 2 Unit of Tropical Diseases, Institute of Tropical Medicine, Antwerp, Belgium; 3 Laboratorium voor Microbiologie, Parasitologie en Hygiëne (LMPH), University of Antwerp, Campus Drie Eiken, Wilrijk, Belgium; 4 Dienst Medische Informatie, Antwerp University Hospital, Edegem, Belgium; 5 Dienst ICT, Antwerp University Hospital, Edegem, Belgium; 6 Department of Virology, Parasitology and Immunology, Faculty of Veterinary Medicine, Ghent University, Merelbeke, Belgium

**Keywords:** taenia, saginata, solium, tapeworm, helminths, Europe, cysticercosis, taeniasis

## Abstract

**Background:**

Few case reports on human infections with the beef tapeworm *Taenia saginata* and the pork tapeworm, *Taenia solium*, diagnosed in Belgium have been published, yet the grey literature suggests a higher number of cases.

**Aim:**

To identify and describe cases of taeniasis and cysticercosis diagnosed at two Belgian referral medical institutions from 1990 to 2015.

**Methods:**

In this observational study we retrospectively gathered data on taeniasis and cysticercosis cases by screening laboratory, medical record databases as well a uniform hospital discharge dataset.

**Results:**

A total of 221 confirmed taeniasis cases were identified. All cases for whom the causative species could be determined (170/221, 76.9%) were found to be *T. saginata* infections. Of those with available information, 40.0% were asymptomatic (26/65), 15.4% reported diarrhoea (10/65), 9.2% reported anal discomfort (6/65) and 15.7% acquired the infection in Belgium (11/70). Five definitive and six probable cases of neurocysticercosis (NCC), and two cases of non-central nervous system cysticercosis (non-CNS CC) were identified. Common symptoms and signs in five of the definitive and probable NCC cases were epilepsy, headaches and/or other neurological disorders. Travel information was available for 10 of the 13 NCC and non-CNS CC cases; two were Belgians travelling to and eight were immigrants or visitors travelling from endemic areas.

**Conclusions:**

The current study indicates that a non-negligible number of taeniasis cases visit Belgian medical facilities, and that cysticercosis is occasionally diagnosed in international travellers.

## Introduction

*Taenia saginata* and *Taenia solium* are the two most common species of tapeworms causing infection in humans. Cattle and pigs are intermediate hosts of *T. saginata* and *T. solium*, respectively. They acquire muscular infection, cysticercosis, upon ingestion of *Taenia* spp. eggs shed with the stools of human tapeworm carriers, either through direct contact or indirectly via contaminated water or application of sewage sludge [1]. Taeniasis, an intestinal infection of humans with the adult tapeworm, is acquired by consuming undercooked infected meat and usually causes only mild clinical symptoms [1], with complications rarely occurring, e.g. intestinal obstruction [2]. In the case of *T. solium*, humans can also acquire cysticercosis upon accidental ingestion of eggs. In humans, the larval stage has a marked affinity for the central nervous system (CNS), causing a condition called neurocysticercosis (NCC). Epilepsy/seizures, chronic headaches and focal deficits are among the most common manifestations of NCC [3]. Globally, *T. solium* was ranked the first food-borne parasite of public health concern [4] and the leading cause of deaths from food-borne diseases [5]. In Europe, *T. solium* was ranked as 10th most important food-borne parasite of public health concern, whereas *T. saginata* was ranked as the 13th [6].

*T. saginata* carriers are common worldwide, including in Europe, yet their number is not well estimated, possibly because of the mild symptoms related to infection [7-11]. In Europe, the infection has been detected both in cattle and humans, suggesting ongoing transmission of the parasite [10-12]. Conversely, the presence of *T. solium* is considered to be restricted mainly to areas with poor sanitary conditions, inadequate hygiene, open defecation, the presence of free roaming pigs and poverty [13]. Human cysticercosis cases are found in vast areas of Africa, Asia and Latin America where *T. solium* is endemic [14]. However, cases have also been reported in non-endemic areas, such as the United States (US), Canada and Europe. Recent reviews describe a total of 275 case reports for western Europe and 58 for eastern Europe for the period 1990 to 2015 [10,11]. Cysticercosis cases diagnosed in these areas often arise from returning travellers and immigrants from endemic areas [15], as well as from untreated *T. solium* tapeworm carriers who would pose a risk to themselves, family members and other contacts in non-endemic areas [16-18]. It is important to obtain accurate epidemiological data on cysticercosis cases in humans and pigs, as well on taeniasis cases caused by *T. solium* in humans. Currently, neither taeniasis nor human cysticercosis are notifiable diseases in the European Union (EU), which limits assessment of the epidemiology of *T. saginata* and *T. solium* in this area [10,11,19].

In Belgium, few reports are available on human *Taenia* spp. infections. Only two taeniasis cases [20,21] and two NCC cases, diagnosed in Belgium, have been published and described in scholarly publications [22,23]. When screening grey literature, however, there are indications that an additional number of taeniasis cases have occurred in Belgium [10]. For instance, between 1980 and 1989, the annual sales of niclosamide doses, a drug prescribed for tapeworm infection, i.e. taeniasis, diphyllobothriosis, hymenolepiosis, fluctuated between 35,000 and 60,000 [24]. In 2013, 11,350 niclosamide doses were sold [25]. Moreover, a review of grey literature indicated that hospital databases and national registries in Europe, including countries neighbouring Belgium, harbour information on a large number of cysticercosis cases diagnosed between 1990 and 2015 that have not been described in scholarly publications (4,901 in western Europe and 772 in eastern Europe) [10,11].

Given the lack of information on the occurrence of taeniasis and human cysticercosis in Belgium, the primary objective of this study was to identify and describe cases of taeniasis and cysticercosis diagnosed in two Belgian referral medical institutions from 1990 to 2015. More specifically, we aimed to summarise the number, socio-demographic information, clinical features, diagnostic test results and treatment of taeniasis and cysticercosis cases.

## Methods

### Study design and setting

This observational study consists of a retrospective analysis of data on suspected and confirmed taeniasis and cysticercosis cases diagnosed between 1990 and 2015 in two Belgian referral medical institutions: the Institute of Tropical Medicine Antwerp (ITMA) and the Antwerp University Hospital (UZA). The ITMA is the national reference centre for tropical medicine and parasitic diseases in Belgium, and runs a large travel clinic. The UZA is a tertiary teaching hospital that hosts the hospitalisation unit of the ITMA. The institutions closely collaborate on the integrated care of patients with tropical diseases. Serology for cysticercosis is performed at the ITMA for patients of both institutions, while stool examination is performed at each institution separately.

### Study population and data sources

The search strategy differed at the two institutes, but aimed for maximal data capture from available sources (Table 1). At the ITMA, the central laboratory database was searched for patients with serological and/or stool analyses positive for *Taenia* spp.. Following this, the medical files of patients positive for any of the tests mentioned were reviewed, additional relevant data were collected, and an ITMA database for suspected taeniasis and cysticercosis cases was created. Only electronic files were searched, which were available from 1994 onwards at ITMA.

**Table 1:** Characterisation of data sources for retrospective data collection of taeniasis and cysticercosis cases diagnosed at two referral medical institutions, Antwerp, Belgium, 1990–2015

Data source	Type of data retrieved	Data availability period
Institute of Tropical Medicine Antwerp (ITMA)		
Central laboratory database	Patients with serology and/or stool examination positive for *Taenia* spp.	1994–2015
Antwerp University Hospital (UZA)		
Central laboratory database	Patients with submitted samples for *Taenia* spp.-related serology Patients with stool examination positive for *Taenia* spp.	1990–2015
Central medical record database	Patients retrieved through keyword query^a^	2001–2015
Uniform hospital discharge dataset	Patients with registered ICD-9 code for taeniasis or cysticercosis (1990–2014) Patients with registered ICD-10 code for taeniasis or cysticercosis (2015)	1990–2015

ICD: International Classification of Disease.

^a^ Tapeworm OR lintworm OR platworm OR taenias* OR tenias* OR (taenia AND (solium OR saginata)) OR (tenia AND (solium OR saginata)) OR neurocystic* OR cysticerc*.

At the UZA, the first step was to search the central laboratory database for patients with samples submitted for *Taenia* spp.-related serology and/or stool examination positive for *Taenia* spp.. Additionally, the following keyword query was run on data for the period 2001 to 2015 in the central medical record database: tapeworm OR lintworm OR platworm OR taenias* OR tenias* OR (taenia AND (solium OR saginata)) OR (tenia AND (solium OR saginata)) OR neurocystic* OR cysticerc*. Hospitals in Belgium are also required to register clinical data of non-ambulatory patients in the government-run uniform hospital discharge dataset (UHDDS). The UZA UHDDS was searched for patients with taeniasis or cysticercosis related International Classification of Disease (ICD)-9 (1990–2014) and ICD-10 diagnosis codes (2015) (see Supplementary Table S1 for full list of codes and description). Medical records of all retrieved patients from these three databases at UZA were reviewed, relevant additional data were collected and a specific UZA database for suspected taeniasis and cysticercosis cases was created after excluding duplicates within each database. The databases of ITMA and UZA were then merged and checked for duplicates, which were excluded from further analysis.

### Case definition

A taeniasis case was considered confirmed when *Taenia* spp. eggs or proglottids were identified upon stool examination, or where an ITMA/UZA physician witnessed an adult *Taenia* spp., e.g. during surgery. As eggs of *Taenia* spp. cannot be distinguished in stool, species identification, i.e. the differentiation of *T. saginata* vs *T. solium,* was based on the number of uterine branches in expelled proglottids when available.

Patients in the cysticercosis database were first listed based on the presence of cysticercosis-related signs or symptoms, being registered with an ICD code for cysticercosis or any mentioning of cysticercosis as part of a differential diagnosis. Subsequently, patients on the list were evaluated by two medical doctors experienced in tropical medicine, and ultimately classified as following: definitive NCC case, probable NCC case (both based on the revised Del Brutto criteria [26]), definitive non-CNS CC case (based on anatomopathology results), unlikely NCC or non-CNS CC case (i.e. medical files indicate atypical symptoms and imaging results), patient with other definitive diagnosis (i.e. medical file mentions final diagnosis different from NCC or non-CNS CC diagnosis), or patient with insufficient information available to allow full evaluation.

### Variables

Variables collected for the taeniasis database were: demographic information, country of origin, travel history (with no restriction in time), stool examination results, clinical presentation, treatment and outcome. Variables collected for the cysticercosis database were: demographic information, travel and immigration history, clinical presentation, results of stool microscopy and antibody- or antigen-based serological tests for *Taenia* spp. as well as for other helminths whenever available (i.e. *Echinococcus* spp., *Fasciola* spp., filariae, *Schistosoma* spp., *Strongyloides stercoralis*, *Toxocara* spp. and *Trichinella* spp.), imaging and treatment.

### Diagnostic tools

At both institutions, stool parasitological analyses are run for *Taenia* spp. detection, i.e. direct microscopic examination and parasitological examination after enrichment.

ITMA conducts the serological analyses for *T. solium* for both institutions. It runs a qualitative *T. solium* IgG antibody (Ab) test using the commercial Cysticercosis Serum Microwell ELISA kit (DRG International Inc., Springfield, New Jersey, US). The manufacturer reports a sensitivity (Se) of 87% (95%CI: 69.3–96.2%) and a specificity (Sp) of 96% (95%CI: 85.7–99.5%), and mentions the possibility of cross-reactions in case of *Echinococcus* spp. infections. Results are expressed as negative or positive. Furthermore, the ITMA performs a *T. solium* antigen (Ag) test using the commercial Cysticercosis Ag ELISA kit (apDia, Turnhout, Belgium), with a reported overall (both viable and dead cysts) Se of 94.0% (95%CI: 87.4–97.8%). In a panel of Peruvian samples from a non-endemic area, the Sp was 100.0% (95%CI: 83.2–100.0%), whereas in a group of Belgian blood donors, the Sp was 99.3% (97.6–99.9%), according to the manufacturer. Results are expressed as negative (optimal density (OD) ≤ 0.8), positive (OD ≥ 1.3) or inconclusive (0.8 < OD < 1.3).

### Data retrieval

**Figure 1:**
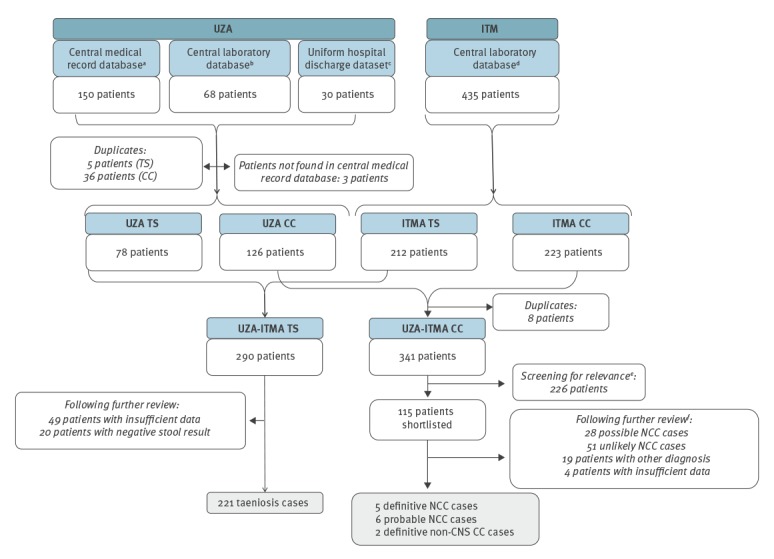
Case retrieval strategy for taeniasis and cysticercosis cases diagnosed at two referral medical institutions, Antwerp, Belgium, 1990–2015

A total of 683 patients were retrieved from the UZA and ITMA databases (Figure 1). After duplicate removal within each institute, taeniasis and cysticercosis databases were created for each institution, and then merged per condition. The merged ITMA-UZA taeniasis database contained data for 290 patients, whereas the merged ITMA-UZA cysticercosis database contained data for 341 patients. Merged databases then underwent a final check for duplicates and critical review of medical files.

CC: cysticercosis; NCC: neurocysticercosis; ITMA: Institute of Tropical Medicine Antwerp: TS: taeniasis; UZA: Antwerp University Hospital.

^a^ Search for patients retrieved through keyword search.

^b^ Search for patients with samples submitted for *Taenia* spp. related serology and/or stool examination positive for *Taenia* spp..

^c^ Search for patients with registered International Classification of Disease (ICD)-9 code (1990–2014) and/or ICD-10 code (2015) for taeniasis or cysticercosis.

^d^ Search for patients with positive serological test results and/or positive stool analysis results.

^e^ Shortlisting based on screening for relevance (i.e. cysticercosis-related signs or symptoms, being registered with an ICD code for cysticercosis or any mentioning of cysticercosis as part of a differential diagnosis).

^f^ Detailed additional review of medical files by two medical doctors with expertise in tropical medicine.

### Merged taeniasis database

In the merged ITMA-UZA taeniasis database (290 patients), 69 patients were excluded on the basis of a negative stool test result (n = 20) or there being insufficient data to evaluate the case further (n = 49). The final dataset contained 221 confirmed taeniasis cases.

Of the eight patients with a taeniasis-related ICD-9 or ICD-10 code registered, only two cases could be confirmed: one had a positive stool test result while an adult *Taenia* spp. was seen and removed during an unrelated laparoscopic procedure for the other. Both cases were added to the dataset. Of the remaining six, one had a negative stool sample analysed and five lacked information on why they were registered as taeniasis cases.

### Merged cysticercosis database

The merged ITMA-UZA cysticercosis database contained 341 potential cysticercosis patients (Figure 1). Medical records of these patients were screened for relevance (i.e. cysticercosis-related symptoms or signs, mentioning of cysticercosis in medical record, or registered ICD code for cysticercosis), with a shortlist of 115 patients resulting. Of these, 102 were excluded after additional detailed review of medical records by two medical doctors with experience in tropical medicine: 28 were categorised as possible NCC cases; 51 as unlikely NCC cases; 19 with different final diagnoses established; and four with insufficient information to render evaluation possible. The final dataset contained five definitive cases of NCC, six probable NCC cases and two definitive cases of non-CNS CC.

Of the 19 patients in the database who had a relevant ICD-9 or ICD-10 code registered, three were definitive NCC cases, three were probable NCC cases, five were categorised as possible NCC cases, two as unlikely NCC cases, two as having different diagnosis and four medical files did not contain sufficient information to allow evaluation.

### Statistical methods

Descriptive statistical analyses, i.e. the calculation of proportion, percentage, median and range were performed in R software version 3.5.1 (R Foundation, Vienna, Austria) [27].

### Ethical statement

For this retrospective analysis, we obtained ethical approval from the Institutional Review Board of ITMA (1018/15) as well as from the UZA Ethics Committee (15/34/350). Both the UZA and the ITMA apply an opt-out strategy for the use of de-identified retrospective medical data. After checking the final databases for duplicates, all names were removed and a study-specific patient code was generated. Moreover, only the variables of interest were extracted for the study analyses and all other information was removed. Because of the low number of cysticercosis cases obtained through our search and to ensure non-identifiability of patients, clinical and laboratory data were presented separately from background variables such as demographic information, country of origin and travel history, and in a different order. Clinical and laboratory data were presented by year of diagnosis, whereas as background variables were presented by age group.

## Results

### Number of samples analysed

The annual number of stool samples analysed at ITMA/UZA for parasites, including *Taenia* spp., for the period 1994 to 2015 fluctuated (median: 5,108; range: 4,150–6,786), with a sharp increase from 4,183 in 2007 to 6,595 in 2008. This was when UZA also started running large numbers of stool examinations. The annual number of serum or cerebrospinal fluid samples analysed with the *T. solium* Ab ELISA gradually decreased over 1994 to 2015, with 1,623 samples analysed in 1994 versus 241 in 2015 (median: 492.5; range: 224–1,623). On the contrary, the annual number of such samples analysed with the *T. solium* Ag ELISA increased from 1998 (n = 46) to 2005 (n = 507), but decreased thereafter to 163 in 2015 (median: 237.5; range: 46–507).

### Taeniasis cases

**Figure 2:**
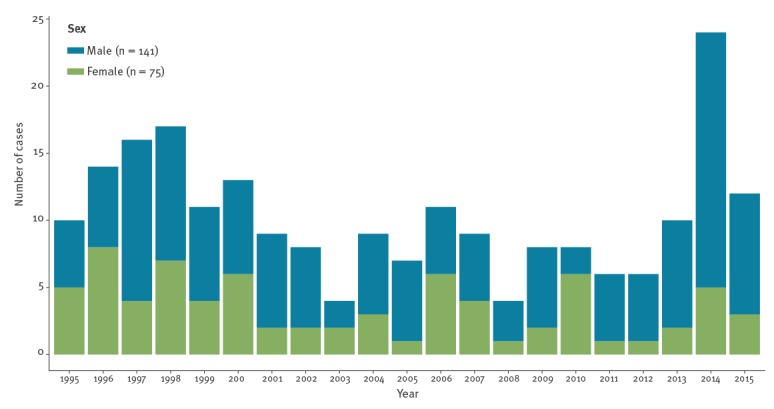
Confirmed taeniasis cases by sex diagnosed at two referral medical institutions, Antwerp, Belgium, 1990–2015 (n = 216^a^)

^a^ Of 221 confirmed taeniasis cases, there were five missing values.

A total of 221 confirmed taeniasis cases were identified. The median number of confirmed taeniasis cases per year was 9, with peaks of 18 in 1998 and 24 in 2014 (Figure 2). The median percentage of confirmed taeniasis cases on the total number of samples analysed for parasites (1995–2015) was 0.21% (range: 0.06–0.35%). For 170 of 221 confirmed taeniasis cases (76.9%), the infection was reported to be caused by *T. saginata*, while for the other cases (23.1%), the species was not mentioned. No cases of taeniasis caused by *T. solium* were identified. The majority of taeniasis cases were male (141/216 (5 missing values (mv)); 65.3%), adult individuals (168/206 (15 mv); 81.6%).

Ten individuals with confirmed taeniasis reported diarrhoea (10/65; 15.6%), six anal discomfort (6/65; 9.2%), five general itch (5/65; 7.7%), eight reported having abdominal pain (8/65; 12.3%) and 26 of 65 were asymptomatic at the time of diagnosis (40.0%) (156 mv).

Treatment information was available for 30 confirmed taeniasis cases. Most cases were treated with praziquantel (n = 19), while others were treated with niclosamide (n = 5), albendazole/mebendazole (n = 2) or a combination of drugs (n = 4), of which three were a combination of niclosamide and praziquantel.

For 70 confirmed taeniasis cases there was information on travel history. Of these, 11 (15.7%) were autochthonous cases who had not travelled outside Belgium in the past 10 years; seven (10.0%) were considered allochthonous cases as they were described as recent immigrants or children who were recently adopted. The remaining 52 cases (72.3%) had travelled but it could not be determined whether the infection was acquired abroad or in Belgium as the dates of travel were not specified.

### Cysticercosis cases

Our search identified five definitive cases of NCC, whereas another six were categorised as probable NCC cases. Two definitive cases of non-CNS CC were also identified. Of the five definitive NCC cases, three were female and two were male, one was below 18 years old, three were between 19 and 30 years old and one was between 31 and 49 years old (Table 2). For the youngest definitive NCC case, no information apart from the anatomopathological result was available. Of the other four definitive cases, two were born in Belgium, one of whom travelled to South America 1 year before the diagnosis and one of whom had travelled to several countries in Asia 3 years before the diagnosis, one had immigrated from sub-Saharan Africa 13 years before the diagnosis was established and one was a visitor from South America. While three had a single lesion detected on magnetic resonance imaging (MRI), their symptoms and signs presented were diverse: one suffered from epilepsy, while the other two reported headaches in combination with different neurological disorders, e.g. balance disorder and dysesthesia (Table 3). The remaining definitive NCC case with imaging information available, in contrast, had 20 lesions, but exhibited no symptoms. The overall time between onset of symptoms and diagnosis for the definitive NCC cases ranged between 0 and 5 months. Of the four definitive NCC cases with serology available only one had positive *T. solium* Ab-ELISA or Ag-ELISA results (positive Ag-ELISA).

**Table 2:** Characteristics of definitive and probable cysticercosis cases diagnosed at two referral medical institutions, Antwerp, Belgium, 1990–2015

Type of cysticercosis case	Age category (years)	Geographical area of origin	Travel/immigration	Geographical area of travel/immigration
Definitive NCC	0–18	NA	NA	NA
	19–30	South America	Travel	Western Europe
	19–30	Sub-Saharan Africa	Immigration	Sub-Saharan Africa
	19–30	Western Europe	Travel	South America
	31–49	Western Europe	Travel	Central/Eastern/Southern Asia
Probable NCC	19–30	Southern Asia	Immigration	Southern Asia
	19–30	Southern Asia	Immigration	Southern Asia
	19–30	Sub-Saharan Africa	Immigration	Sub-Saharan Africa
	31–49	Eastern Asia	Immigration	Southern Asia
	31–49	Southern Asia	Immigration	NA
	31–49	Sub-Saharan Africa	Immigration	NA
Definitive non-CNS CC	31–49	NA	NA	NA
	31–49	NA	NA	NA

CC: cysticercosis; CNS: central nervous system; NA: not available; NCC: neurocysticercosis.

Cases were ordered by age category and then alphabetically by region of origin.

**Table 3:** Definitive neurocysticercosis cases diagnosed at two referral medical institutions, Antwerp, Belgium, 1990–2015

Year of diagnosis at ITMA or UZA	Clinical symptoms	Serology	Stool	Imaging	Additional information
2001	None	1 month before diagnosis and at diagnosis: Ab-ELISA: neg; Ag-ELISA: pos 1 month after diagnosis: Ag-ELISA: neg	NA	MRI: 20 lesions, with ring enhancement, no or slight oedema 2 months after diagnosis: MRI: one lesion, several patchy zones	NA
2004	NA	NA	NA	NA	NA
2009	Epilepsy	Ab-ELISA: neg	Neg	MRI: 1 lesion temporo-occipital right, perilesional oedema 1 month after diagnosis: MRI: reduction in volume and perilesional oedema	No eosinophilia *Taenia solium* Ab/Ag-ELISA CSF: neg
2010	6 months before diagnosis: photopsia left eye, balance disorder, mild headache	Ab/Ag-ELISA: neg	NA	1 month before diagnosis: MRI: one cystic ring-enhancing lesion occipital horn right, perilesional oedema, one presumed vascular lesion frontal right	NA
2011	1 month before diagnosis: headache, dysesthesia, nausea	Ab/Ag-ELISA: neg	NA	CT, MRI: one cystic lesion fronto-parietal left, perilesional oedema	No eosinophilia

Ab: antibody; Ag: antigen; CSF: cerebrospinal fluid; CT: computed tomography; ITMA: Institute of Tropical Medicine Antwerp; MRI: magnetic resonance imaging; neg: negative; NA: not available; pos: positive; UZA: Antwerp University Hospital.

Of the four definitive NCC cases with treatment information available, two received anthelmintic treatment, one underwent surgery and one was treated with a combination of anthelmintics and surgery. Anthelmintic treatment consisted of albendazole for three cases, and both praziquantel and albendazole for one case.

In the group of probable NCC cases (n = 6), one was female and five were male (Table 2). Two cases came from the Democratic Republic of the Congo, while the others were from India, China (Tibet), Nepal and Afghanistan.

One probable NCC case had a single lesion, one had two lesions, three had three or more lesions, and for one probable case, this information was not available (Table 4). Symptoms and signs present in the group of probable NCC cases included epilepsy, headache and other neurological disorders, e.g. partial paralysis. The time between onset of symptoms and diagnosis for the probable NCC cases ranged between 2 months and ca 16 years.

**Table 4:** Probable neurocysticercosis cases diagnosed at two referral medical institutions, Antwerp, Belgium, 1990–2015

Year of diagnosis at ITMA or UZA	Clinical symptoms	Serology	Stool	Imaging	Additional information
2006	1 year before diagnosis and at diagnosis: epilepsy	NA	NA	MRI: several cystic lesions, frontal cyst with inflammation	NA
2011	3 years before diagnosis: intermittent headaches, insomnia	10 and 8 months before diagnosis: Ab-ELISA: pos; Ag-ELISA: neg 7 months before diagnosis: Ab-ELISA: neg At diagnosis: Ab-ELISA: pos	8 months before diagnosis: neg	MRI: cystic enhancing lesion in nucleus caudatus, perilesional oedema, superior two smaller cystic lesions 5 months after diagnosis: MRI: reduced oedema	10 and 9 months before diagnosis: *Schistomosa* ELISA: pos; *Strongyloides*-ELISA: pos; ELISA for filaria: pos 8 months before diagnosis: eosinophilia At diagnosis: *Schistosoma*-ELISA: pos; *Strongyloides*-ELISA: pos
2012	Prior to the diagnosis: epileptic seizure Around 4 months before diagnosis: sensory disorders	Ab-ELISA: neg, Ag-ELISA: pos	NA	NA	NA
2012	3 years before diagnosis: four epilepsy events, thereafter no symptoms	Ab/Ag-ELISA: neg	Neg	2 months before diagnosis: CT: intracranial lesions At diagnosis: MRI: five supratentorial lesions: three with ring enhancement, two temporal right, one occipital right, one frontal right, one left parietal	Accidental finding ELISA for filariae: pos; *Strongyloides*-ELISA: pos
2012	8 and 6 years before confirmatory diagnosis at ITMA or UZA^a^: epilepsy At diagnosis: twitching feeling in foot, chronic headache	NA	NA	CT: normal; MRI: one lesion cortical frontal right	NA
2012	Since 3 years before diagnosis: chronic headache 3 months before diagnosis: paresis left leg	2 months before diagnosis: Ab/Ag-ELISA: neg	NA	MRI: two nodular lesions parietal cortex right	2 months before diagnosis: eosinophilia; *Strongyloides* PCR and ELISA: pos 1 month before diagnosis: *Strongyloides* PCR and ELISA: neg At diagnosis: no eosinophilia

Ab: antibody; Ag: antigen; CT: computed tomography; ITMA: Institute of Tropical Medicine Antwerp; MRI: magnetic resonance imaging; neg: negative; NA: not available; pos: positive; UZA: Antwerp University Hospital.

^a^ Diagnosis earlier established outside of ITMA or UZA.

One probable NCC case had a positive *T. solium* Ag-ELISA result only, while another had a positive Ab-ELISA result only. No cases had eosinophilia, except for two with documented concurrent helminth infection.

No information on treatment was available for one probable NCC case, whereas four received an anthelmintic treatment with albendazole and one underwent surgery. One of the probable NCC cases was an accidental finding during imaging for an unrelated indication.

Both cases of definitive non-CNS CC, one diagnosed in 2005 and one in 2008, had a single nodule in the abdominal skin. For the latter, the nodule was reported to have developed 13 years earlier. No further information was available for these patients.

## Discussion

We conducted a comprehensive retrospective investigation for taeniasis and cysticercosis cases in two referral medical institutions in Belgium. The total number of confirmed taeniasis cases retrieved for the study period was higher than those extracted from hospital/laboratory-based registries from other western European countries, e.g. France, Denmark, Portugal, but the median annual number of cases was lower than that reported in the epidemiological bulletins and national registries of the United Kingdom, Spain and Slovenia [10]. Confirmed taeniasis cases in our study reported rather mild symptoms, which is in line with the literature [1]. All taeniasis cases for whom the causative species could be identified were *T. saginata* carriers. Eleven of 70 taeniasis cases were acquired in Belgium. Cattle acquire bovine cysticercosis through ingestion of eggs shed by human *T. saginata* carriers, with research in Belgium pointing to wastewater contaminating pastures as a source of infection [28]. A recent study estimated that over 33% of Belgian cattle may be infected with *T. saginata* [29], suggesting the continued completion of the parasite’s lifecycle in the country. Given this and the habit of eating of raw or undercooked beef in Belgium, a certain risk of acquiring taeniasis remains. The continued transmission of *Taenia saginata* in Belgium has an economic impact: taeniasis has an estimated cost for the human health sector of up to EUR 795,858 per year, whereas bovine cysticercosis has an estimated cost for the meat industry of up to EUR 3,408,455 per year [30]. Furthermore, for 23.1% of taeniasis cases in our study, the causative species was not known and this group could thus potentially include some *T. solium* carriers. They would pose a risk to themselves, family members and other contacts with respect to cysticercosis development [16-18]. Unfortunately, classical methods to examine stool cannot always distinguish species and molecular differentiation, although recommended, is not routinely done in Europe [10,11].

The number of definitive and probable cysticercosis cases found in our study was in line with the number of cases reported at the hospital/laboratory level in Austria, Denmark and Sweden, but much lower than the number of cases for Portugal, Spain the Netherlands, France and Italy [10]. Most cases seemed to be detected closer towards the end of the study period, possibly because of an actual increase in cases or increased awareness of specialists at the study institutes about the condition. After 2015, cysticercosis continued to be diagnosed at both institutes, with another three definitive NCC cases reported (Supplementary Table S2, Supplementary Table S3). Overall, the definitive and probable cysticercosis cases identified in our study had diverse travel, migration and age characteristics, indicating that identifying high-risk patients for NCC is difficult. For some cases, it took several months and even years before the diagnosis was established, possibly because of the often non-specific signs and symptoms presented by patients, e.g. chronic headaches, and limited experience of some physicians with tropical diseases. Furthermore, serological test results are not always conclusive. For instance, commercially available Ab-ELISA kits are reported to exhibit low sensitivity and frequent cross-reactions [31], and while the enzyme-linked immunoelectrotransfer blot (EITB) assay has a close to perfect performance in terms of sensitivity and specificity to detecting Ab, the test is expensive, cumbersome and not routinely used [32]. Good sensitivity and specificity were reported for the Ag-ELISA; but this test only detects the presence of viable cysticerci, the earliest stage of NCC [33]. NCC-associated epilepsy however, is thought to occur when cysticerci present in the CNS start to degenerate or have even calcified [34,35]. Current diagnostic guidelines for NCC therefore advise the combined evaluation of imaging results, clinical manifestations and exposure-related factors, e.g. serology, travel history, to establish the diagnosis and assess the degree of certainty [26].

This study has several limitations. At the ITMA, software did not allow searching the medical records for certain terms or ICD codes, which means that a certain number of true cases may have been missed. However, because UZA medical records were searched for specific terms and because the ITMA and UZA databases were merged, the risk of missing true cases was rather low. In contrast, relying on positive Ab-based serology as one of the search criteria may have led to the inclusion of false-positive cases because of cross-reactions. However, the thorough critical review of medical files allowed excluding irrelevant cases. It is noteworthy, that the use of ICD codes to retrieve taeniasis and cysticercosis cases was not faultless as some cases with relevant ICD codes could not be assigned undoubtedly to taeniasis or cysticercosis diagnosis and therefore had to be excluded. Furthermore, as is inherent to retrospective surveys of medical files, information was often incomplete and a critical review of imaging results was not possible when they were not electronically stored. Finally, as case identification requires the intensive review of medical files and related ethical clearance, our study was restricted to two Belgian referral medical institutions. Nevertheless, we expect most cysticercosis cases to have received a confirmatory diagnosis at ITMA, as it is the national reference national reference centre for infectious and tropical diseases. As for the taeniasis cases, apart from dedicated screening in adopted children and recent immigrants from endemic areas at ITMA and UZA, other large hospitals in Belgium are expected to have diagnosed a considerable number of cases as well during the study period.

Overall, the findings of the current study confirm that taeniasis and cysticercosis cases are consulting Belgian hospitals in larger numbers than reported in scholarly publications. As a proportion of taeniasis cases caused by *T. saginata* were acquired in Belgium, improved taeniasis case management, including correct treatment of cases and disposal of expelled tapeworms, as well as a multi-sectoral One Health approach are warranted to control the parasite’s transmission in the country. Furthermore, molecular differentiation of tapeworms is advised in order to detect *T. solium* carriers. Regarding cysticercosis, clinical awareness as well as serological testing of individuals at risk, such as travellers and immigrants, with suggestive symptoms are key. Also the complexity of management should be highlighted during medical training to ensure adequate referral to, or supervision by experts in the field such as those in tropical medicine.

## Supplementary Data

151000 Supplementary Material
