# Supplementary material for: Cysticercosis and taeniasis cases diagnosed at two referral medical institutions, Belgium, 1990 to 2015
Source: Euro Surveill. 2019 Aug 29;24(35):1800589. doi: 10.2807/1560-7917.ES.2019.24.35.1800589 (PMC6724463; doi:10.2807/1560-7917.ES.2019.24.35.1800589)
Supplement: Supplementary Material [file 1800589_DERMAUW_SupplementaryMaterial.pdf]

Supplementary material

"This supplementary material is hosted by *Eurosurveillance* as supporting information alongside the article "Cysticercosis and taeniasis cases diagnosed at two referral medical institutions in Belgium, 1990 to 2015" on behalf of the authors who remain responsible for the accuracy and appropriateness of the content. The same standards for ethics, copyright, attributions and permissions as for the article apply. *Eurosurveillance* is not responsible for the maintenance of any links or email addresses provided therein."

SUPPLEMENTARY TABLE S1. ICD-9 and ICD-10 codes used for retrospective data collection of taeniasis and cysticercosis cases diagnosed at two referral medical institutions, Belgium, 1990-2015

**ICD-9 codes with clarification**
123.0 *Taenia solium* infection, intestinal form
123.1 Cysticercosis
123.2 *Taenia saginata* infection
123.3 Taeniasis, unspecified
**ICD-10 codes with clarification**
B68 Taeniasis
B68.0 *Taenia solium* taeniasis
B68.1 *Taenia saginata* taeniasis
B68.9 Taeniasis, unspecified
B69 Cysticercosis
B69.0 Cysticercosis of central nervous system
B69.1 Cysticercosis of eye
B69.8 Cysticercosis of other sites
B69.81 Myositis in cysticercosis
B69.89 Cysticercosis of other sites
B69.9 Cysticercosis, unspecified

Source: https://www.icd10data.com/

SUPPLEMENTARY TABLE S2. Background of definitive neurocysticercosis cases diagnosed at two referral medical institutions, Antwerp, Belgium, 2017[a]

Age category (years)	Geographical area of origin	Travel/immigration	Geographical area of travel/immigration
31-49	Southern Asia	Immigration	Western Europe
31-49	Western Europe	Travel	Caribbean, Eastern/Western Africa
≥50	Western Europe	Travel	Northern Africa, South-Eastern Asia

Cases were ordered by age category and then alphabetically by geographical area of origin

[a] These cases occurred outside of the original study period of 1990–2015.

SUPPLEMENTARY TABLE S3. Additional definitive neurocysticercosis cases diagnosed at two referral medical institutions, Belgium, 2017[a]

Year of diagnosis at ITM or UZA	Clinical symptoms	Serology	Stool	Imaging	Additional information
2017	Epilepsy, paresis left arm and hand	Ab-ELISA: neg, Ag-ELISA: inconclusive	NA	MRI: single cystic cerebral lesion	No eosinophilia
2017	2 years before diagnosis: left arm paresis; epilepsy	5 months after diagnosis: Ab/Ag ELISA: neg	neg	MRI: single granuloma/calcified lesion with perilesional oedema	2 years before diagnosis at ITM/UZA: diagnosis established outside ITM/UZA; 5 months after diagnosis: no eosinophilia
2017	23 months before diagnosis: epilepsy; 5 episodes afterwards	Ab/Ag-ELISA: neg	neg	18 months before diagnosis: MRI: several ring enhancing lesions; 8 months before diagnosis: MRI and CT: 2 enhancing cystic lesions with perilesional oedema; 2 months before diagnosis: MRI and CT: residual calcifications	18 months before diagnosis at ITM/UZA: diagnosis established outside ITM/UZA; at diagnosis: no eosinophilia

Ab: antibody; Ag: antigen; CT: computed tomography; ITM: Institute of Tropical Medicine Antwerp; MRI: magnetic resonance imaging; neg: negative; NA: not available; pos: positive; UZA: Antwerp University Hospital.

[a] These cases visited ITM/UZA outside of the original study period of 1990–2015.
